# Develop of endocavitary suction device for MiECC on minimally invasive mitral valve surgery

**DOI:** 10.1186/s13019-024-02678-y

**Published:** 2024-03-27

**Authors:** Ignazio Condello, Giuseppe Speziale, Giuseppe Nasso

**Affiliations:** Department of Cardiac Surgery, GVM Care & Research, Anthea Hospital, Via Camillo Rosalba 35/37, Bari, 70124 Italy

**Keywords:** Minimal Invasive Mitral valve surgery, Minimally invasive extracorporeal circulation, Endo-cavitary suction device, Minithoracotomy

## Abstract

The minimally invasive extracorporeal circulation (MiECC) system was developed to minimize the contact of blood with air and foreign surfaces during conventional cardiopulmonary bypass (CPB). It is also aimed to reduce the inflammatory response by further increasing the biocompatibility of the components that make up the MiECC circuits. The Minithoracotomy (MTH) approach for mitral valve disease remains associated with prolonged operative times, but it is beneficial in terms of reduced postoperative complications (renal failure, atrial fibrillation, blood transfusion, wound infection), length of stay in intensive care unit (ICU) and in hospitalization, with finally a reduction in global cost. Combining the use of the MiECC technique with minimally invasive mitral valve surgery (MIMVS) could open up new research scenarios. Although considerable progress has been made in the standardization of the surgical technique, limitations remain to be filled in the setting of Endo-cavitary aspiration for the association of MiECC with MIMVS. In this paper we introduce invention refers to a device and an air-closed endocavitary aspiration system for cardiac chamber surgery, as well as a method aimed at eliminating gaseous micro-embolic activity, hemolysis and CO_2_ aspiration and alteration of carbon dioxide production (VCO_2_) the parameters for goal directed perfusion. The system allows the surgery of the cardiac chambers to be associated with a minimally invasive extra-corporeal circulation circuit.

## Introduction

The Minimally Invasive Extracorporeal Circulation (MiECC) system was innovated to minimize blood contact with air and foreign surfaces during conventional CPB, aiming to reduce inflammatory responses by enhancing biocompatibility. Integrating MiECC with Minimally Invasive Mitral Valve Surgery (MIMVS) opens new research avenues. While progress in standardizing surgical techniques is evident, challenges persist in Endo-cavitary aspiration when associating MiECC with MIMVS [1, 2]. In mitral valve surgery, managing bleeding, especially from the left heart's pulmonary veins, relies on aspirators that suction blood upon air exposure. Intracavitary aspiration of air and blood often requires high negative suction pressures, impacting the surgical field. Filters and open extracorporeal circuits can lead to gas micro-emboli, hemolysis, inflammation, and coagulation issues. This contrasts with closed systems of MiECC, which have a lesser impact on hemodilution, hemolysis, and inflammation [3, 4]. Despite advancements in surgical techniques, overcoming limitations in endocavitary aspiration is crucial when combining MiECC with Minimally Invasive Mitral Valve Surgery (MIMVS). In perioperative practice, a suction cup in the left pulmonary veins maintains a clear surgical field, but it involves aspirating air mixed with blood. Balancing pressure and suction efficiency pose challenges in MiECC management, potentially causing gas micro-emboli, hemolysis, inflammation, and coagulation alterations [2]. In this context we propose a new device for endo-cavitary aspiration to contain and mitigate these items.

### The state of the art in endo-cavitary suction

The state of the art in endo-cavitary suction for mitral valve surgery is currently addressed by the gold standard, which involves the use of an aspirator known as SUMP [2]. This device, utilized in both conventional (sternotomy) and minimally invasive (mini-thoracotomy or thoracoscopy) approaches, tends to suction blood mixed with air in a swirling and disorganized manner from the pulmonary veins [3, 4]. This process can lead to increased hemolysis and the generation and transport of gaseous micro-embolic activity. Additionally, the aspiration of CO_2_ from the surgical field compromises the VCO_2_ parameter in metabolic monitoring of extracorporeal circulation, potentially contributing to acute kidney injury [5, 6]. Consequently, the challenge persists in developing an endo-cavitary aspiration device that can eliminate air-blood contact and address the pathophysiological alterations associated with conventional closed systems of extracorporeal circulation.

## Materials and methods

In the initial phase of developing the project for minimally invasive mitral valve surgery to prevent blood-air contact and avoid CO_2_ suction, we began using the 13 Fr Retrograde Cannula, Manual Inflate, Silicone Body (Medtronic,Minneapolis, MN). This cannula is validated and certified for retrograde perfusion of the coronary venous sinus in one or more pulmonary veins (up to two). However, despite its effective functionality, we encountered limitations related to the hole shape, small cannula diameter, and potential visual clutter on the surgical field [7, 8]. In response to these limitations, we designed a new device for endo-cavitary aspiration and an accompanying system. This system, featuring the inflation of at least one perforated balloon inside a venous lumen, completely occludes the venous lumen, allowing blood to pass towards a hollow tubular suction element through holes on the balloon (Patent pending). Compared to existing devices, our design includes a hollow tubular element with an elongated helical shape, configured to aspirate fluids during surgical operations. The helical structure enables the management of laminar flow, minimizing turbulence and providing control over excess negative pressure (or vacuum) upstream of the device. This prevents the collapse of the venous lumen wall around the balloon, which, if anchored to the pulmonary venous lumen, could impede venous blood aspiration. Moreover, our device, in contrast to the sump (gold standard) or spring aspirator, isolates the pulmonary veins with a controlled bleeding rate through the inflation of a polyurethane balloon. This eliminates the mixed vortex regime with air and CO2, leading to a reduction in micro-embolic activity, hemolysis, and the aspiration of CO2 administered in the surgical field within the extra-corporeal circuit [5, 8, 9].

## Components of the endo-cavitary suction device

A device for endo-cavitary aspiration comprises:

- an elongated, first flexible tubular element with a closed proximal end, featuring multiple holes,
- a hollow tubular element with an elongated helical shape connected to the distal end of the first tubular element, designed for suction,
- at least one perforated inflatable balloon,
- at least one elongated, flexible hollow tubular element passing through one or more holes in the closed proximal end, connected to the balloon for inflation or deflation via fluid introduction,
- another elongated, flexible hollow tubular element passing through one or more holes in the closed proximal end, connected to the balloon and a hollow tubular suction element passing through the first element for suction,

The device is distinguished by the inflatable balloon, which, when positioned within a venous lumen and inflated, completely occludes the venous lumen, permitting blood passage toward the hollow tubular suction element through the balloon's holes.

**Fig. 1:**
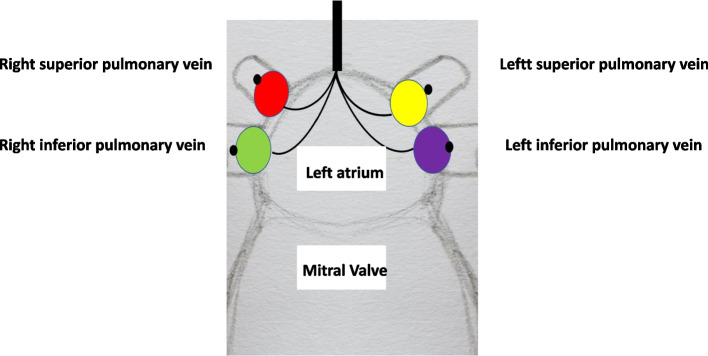
Endo-cavitary suction device application on pulmonary vein

**Fig. 2:**
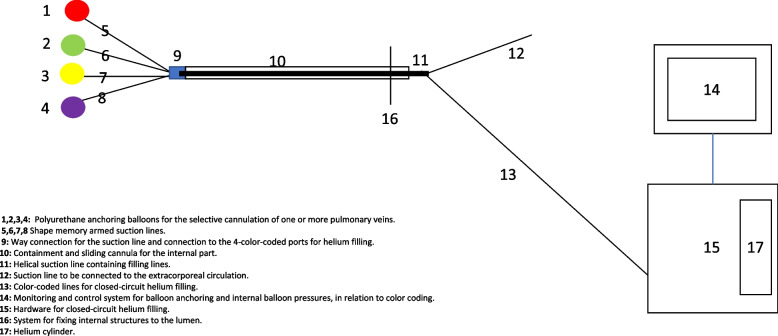
Endo-cavitary suction device sketch, design and component description

An endo-cavitary aspiration system includes the device, a controller for managing balloon filling and internal pressure, an extracorporeal circulation device, and a vacuum generation device for aspiration. Additional advantages and features are detailed in the description (Figs. 1 and 2).

### Function and application

In one configuration, multiple balloons, preferably at least 1,2, 3, or 4, can be assembled onto the suction line within the first element either before or during the operation, depending on the desired anchoring quantity to the pulmonary veins (Fig. 1). Blood entering the balloon holes is directed into the suction line, enabling selective anchoring based on visual bleeding and the severity of venous return on the pulmonary veins. The elliptical-shaped balloon, with a filling volume of 1 to 10 ml (preferably 5 ml), generates a pressure of 150 to 180 mmHg when inflated, ensuring a hermetic blood seal within the venous lumen, aiding in both aspiration and physically isolating bleeding for optimal surgical field visualization. In this embodiment, the balloon, the element connected to it, and the hollow tubular suction element passing through the first element are constructed from a polymeric material, preferably polyurethane, known for its high pressure resistance, biocompatibility, and sustainability. The balloon's internal pressure, when inflated, achieves 150 to 180 mmHg in the pulmonary venous lumen, maintaining a hermetic blood seal and promoting bleeding isolation for enhanced visualization. The balloon design includes three medial holes and a central hole, strategically placed to facilitate blood aspiration. Different hole numbers and arrangements are possible, but homogeneous blood aspiration management is preferable. To aid identification, each balloon and its associated filling/deflating line are color-coded.

The fluid delivery device for helium administration and pressure monitoring comprises a helium cylinder, a management monitor, and a hardware system providing closed-circuit helium delivery to the balloons based on the color codes, as specified by the surgeon. Additionally, the element connected to the balloon and the hollow tubular suction element may incorporate an internal metal shape memory structure, optimizing space usage during device use without the need for cumbersome syringes. Nitinol, a shape memory alloy, is a suitable material for this purpose.

The element connected to the balloon and the hollow tubular suction element have a diameter of about 0.3 mm to 3.3 mm, preferably 1.67 mm (1 to 10 Fr, preferably 5 Fr). The first flexible tubular element has a diameter of about 3.3 mm to 10 mm, preferably 6.7 mm (10 Fr to 30 Fr, preferably 20 Fr). A fixing system is included inside the first element in this device configuration (Fig. 2).

### Suction management

The present invention introduces a suction device known as the "Roller pump for endo-cavitary aspiration," employing a roller mechanism to generate the required vacuum for endo-cavitary aspiration within the realm of "Extracorporeal Circulation Techniques." These techniques involve temporarily diverting blood from the body through a pump system, oxygenating it via a blood oxygenation unit, such as a membrane oxygenator. Typically utilized in conventional extracorporeal circulation processes, the roller pump manages suction [10]. Additionally, the device allows for the incorporation of "Venturi effect devices," exploiting Bernoulli's principle to create a vacuum in a fluidic system. The Venturi effect is based on the relationship between fluid velocity and pressure, utilizing a duct with narrowing and widening sections. This effect can be harnessed to either suction or push fluids depending on the application. In the context of venous return, the Venturi effect device can aspirate blood or other fluids from the venous system [11]. Furthermore, the present invention introduces the concept of a "negative pressure centrifugal pump," utilizing centrifugal force to propel fluid through the system. The term "negative pressure" signifies the creation of a vacuum in the venous line due to the negative pressure effect generated by the centrifugal pump, occurring prior to the conventional venous bubble trap [12].

## Future perspective and development

The development process for an endo-cavitary suction device for MiECC on minimally invasive mitral valve surgery involves several key stages. Initially, thorough market research and needs assessment are conducted to identify existing gaps and requirements. Subsequently, a multidisciplinary team of engineers, surgeons, and researchers collaborates to conceptualize the device, outlining its key features and functionalities. The design phase follows, incorporating feedback from medical professionals to ensure ergonomic and surgical integration. Prototyping then takes place, involving iterative testing and refinement to optimize the device's performance and usability. Regulatory compliance and adherence to medical standards are crucial considerations throughout this phase. Upon successful prototyping, the manufacturing process commences, involving precision engineering and quality control measures. Rigorous testing, both in simulated environments and real-world surgical scenarios, helps validate the device's safety and efficacy. Collaboration with regulatory bodies is essential to obtain necessary approvals. Finally, the device is introduced to the market through targeted medical conferences and educational initiatives. Continuous feedback loops are established to monitor post-market performance and identify opportunities for further improvement. This comprehensive development process ensures the endo-cavitary suction device meets the highest standards of innovation, safety, and effectiveness in the context of minimally invasive mitral valve surgery.

## Data Availability

No datasets were generated or analysed during the current study.

## Abbreviations

AKI: Acute kidney injury
CPB: Cardiopulmonary bypass
DO_2_: Oxygen delivery
VCO_2_: Carbon dioxide production.
MiECC: Minimally invasive extra-corporeal circulation
MIMVS: Minimally invasive mitral valve surgery
MTH: Minithoracotomy
ICU: Intensive care unit
